# Genomic diversity and novel sequence types of *Pasteurella multocida* from diverse animal hosts in Connecticut, United States, 2022–2023

**DOI:** 10.3389/fvets.2026.1920717

**Published:** 2026-08-17

**Authors:** Jillian Barron, Nina Francesca Soriano, Zeinab H. Helal, Ji-Yeon Hyeon, Guillermo R. Risatti

**Affiliations:** 1 Connecticut Veterinary Medical Diagnostic Laboratory, Department of Pathobiology and Veterinary Science, College of Agriculture, Health and Natural Resources, University of Connecticut, Storrs, CT, United States; 2 Department of Pathobiology and Veterinary Science, College of Agriculture, Health and Natural Resources, University of Connecticut, Storrs, CT, United States; 3 College of Veterinary Medicine, Konkuk University, Seoul, Republic of Korea

**Keywords:** animal hosts, antibiotic resistance, genomic diversity, *pasteurella multocida*, sequence type, whole-genome sequencing

## Abstract

*Pasteurella multocida* is a zoonotic pathogen infecting a wide range of domestic and wild animals. Although genomic data are available for several domestic hosts, isolates from wildlife remain underrepresented. Here, we performed whole-genome sequencing of nine *P. multocida* isolates recovered in Connecticut between 2022 and 2023 from cows, a deer, a rabbit, a cat, and a pheasant to characterize their genetic features and enhance our understanding of the host-associated diversity. All isolates except one feline strain shared a common genotype across multiple host species, being classified as capsular type A and LPS type L3. In contrast, the feline isolate lacked a detected capsule locus and was classified as LPS type L1. Bovine isolates exhibited higher antimicrobial resistance, including the only multidrug-resistant isolate, and exclusively harbored antimicrobial resistance (AMR) genes [*aph*(3′)-Ia, *aph*(6)-Id, *sul2*, and *tet*(H)], whereas non-bovine isolates showed low resistance and no evidence of AMR determinants. Multilocus sequence typing revealed that all bovine isolates belonged to ST1 (Multi-Host scheme) and ST79 (RIRDC scheme), while non-bovine isolates displayed greater genetic diversity, including five novel sequence types. Virulence-associated genes were largely conserved across hosts. Core-genome SNP-based phylogenetic analysis revealed largely host-associated clustering, with bovine isolates grouping with bovine lineages from other countries and non-bovine isolates clustering with genetically diverse international lineages, including avian and human-associated strains. Collectively, these findings highlight host-linked patterns in AMR and genomic diversity of *P. multocida* and underscore the importance of integrated phenotypic and genomic surveillance across animal hosts.

## 1 Introduction

*Pasteurella multocida* is a Gram-negative bacterium with a broad range of hosts and global distribution. It is a zoonotic pathogen responsible for significant morbidity and mortality in both animals and humans (1, 2). It can disseminate across geographic regions and infects a wide spectrum of domestic and wild animals, including poultry, pigs, cattle, buffaloes, rabbits, small ruminants, cats, dogs, and diverse wildlife species (3). In livestock and poultry, *P. multocida* is the etiological agent of several economically important diseases, such as hemorrhagic septicemia in ungulates, atrophic rhinitis in pigs, fowl cholera in birds, snuffles in rabbits, and enzootic pneumonia and shipping fever in ruminants (3). In humans, it primarily causes opportunistic infections associated with animal bites or scratches, although respiratory infections and sporadic septicemia have also been reported (2).

Given its wide host range and pathogenic versatility, *P. multocida* is an important pathogen of veterinary and public health concern. Although the genome sequences of *P. multocida* isolates have been reported in the United States, comprehensive genomic data describing isolates from diverse animal hosts are still limited, and isolates circulating in Connecticut have not yet been characterized at the genomic level. Addressing these gaps is essential for improving our understanding of the population structure and host-associated diversity of this pathogen at a regional scale. In this study, we performed whole-genome sequencing of *P. multocida* isolates obtained from cows, rabbits, deer, cats, and pheasants in Connecticut to characterize their genetic features and enhance our understanding of the host-associated diversity.

## 2 Materials and methods

### 2.1 Study isolates

Nine *P. multocida* isolates from animal tissue samples were subjected to antimicrobial susceptibility tests and whole-genome sequencing (WGS). The isolates were obtained from milk samples from domestic cows (*n* = 5), lung tissues from a wild white-tailed deer (*n* = 1), a domestic cat (*n* = 1), and a domestic rabbit (*n* = 1), and spleen tissue from a domestic ring-necked pheasant (*n* = 1) in the Connecticut Veterinary Medical Diagnostic Laboratory (CVMDL), Department of Pathobiology and Veterinary Science, University of Connecticut, between 2022 and 2023 (Table 1). The BIOLOG MicroLog3 Microbial Identification System (Biolog, USA) was used to identify the species of all isolates.

**Table 1:** Host information, genotypes, and antimicrobial resistance gene profiles of the *P. multocida* isolates sequenced in this study.

Isolate	Organ	Host	Collection date	Diagnosis	Capsular type	LPS	Sequence type		Antimicrobial resistance gene			
							Multi-host MLST	RIRDC MLST	*aph*(3′)-Ia	*aph(6)-Id*	*sul2*	*tet*(H)
22-287	Lung	Deer	2/10/22	Severe necrotizing fibrinopurulent bronchopneumonia	A	L3	374^*^	622^*^	–	–	–	–
22-2477	Milk	Cow	6/13/22	Mastitis	A	L3	1	79	+	+	+	+
22-2831	Lung	Cat	7/14/22	Pulmonary adenocarcinoma with cardiac and renal metastasis	No capsule locus	L1	375^*^	171	–	–	–	–
22-4189	Spleen	Pheasant	11/7/22	Gram-negative septicemia	A	L3	373^*^	623^*^	–	–	–	–
23-11481	Milk	Cow	5/26/23	Mastitis	A	L3	1	79	+	+	+	+
23-11920	Milk	Cow	6/30/23	Mastitis	A	L3	1	79	+	+	+	+
23-11977	Milk	Cow	7/7/23	Mastitis	A	L3	1	79	+	+	+	+
23-973	Lung	Rabbit	7/25/23	Interstitial pneumonia with pulmonary edema and congestion	A	L3	312	519	–	–	–	–
23-12301	Milk	Cow	8/15/23	Mastitis	A	L3	1	79	+	+	+	+

^*^Novel STs submitted and registered in the PubMLST database.

### 2.2 Antimicrobial susceptibility testing

The antimicrobial susceptibility of *P. multocida* isolates was determined using the Kirby-Bauer disk diffusion test with clinically relevant antimicrobial agents for which Clinical and Laboratory Standards Institute guidelines VET01S interpretive criteria are available (4).

### 2.3 Whole-genome sequencing

For WGS, genomic DNA was extracted from pure cultures of *P. multocida* using the DNeasy Blood and Tissue kit (Qiagen, USA) according to the manufacturer's instructions. Sample DNA concentrations were determined using the Qubit dsDNA HS Assay Kit (Invitrogen, USA). Sample libraries were prepared using the Illumina DNA Flex Library Prep Kit (Illumina, USA), followed by dilution to 2 nM. The samples were sequenced using a MiSeq Reagent Kit V2 (500 cycles) cartridge (Illumina) after loading 600 μL of the 10 pM pooled libraries.

### 2.4 Whole-genome sequence analysis

Raw sequencing reads were adapter-trimmed to remove the Illumina adapters and quality-trimmed using BBDuk (Q > 20, minimum length > 50) (https://sourceforge.net/projects/bbmap). The trimmed reads were *de novo* assembled using SPAdes 3.15.5 (5) and default parameters. Assembled contigs with coverage below 5 × and lengths shorter than 300 bp were excluded from the downstream analyses. Genome assembly quality was assessed using QUAST v5.3.0., as implemented on the Galaxy platform (https://usegalaxy.org/). Assembly metrics, including the number of contigs, N50, total length, largest contig, and GC content were summarized in Supplementary Table 2.

Multilocus sequence typing (MLST) was performed using both the Multi-Host and RIRDC *P. multocida* MLST schemes in the PubMLST database. Isolates with previously unassigned sequence types (STs) were registered in the PubMLST database. Virulence-associated genes as well as capsular and lipopolysaccharide (LPS) genotypes were screened using ABRicate version 1.0.1 (https://github.com/tseemann/abricate) with a custom database derived from a previous study (6). For strains with untypable capsular genotypes, multiplex capsular PCR was performed as described in a previous study (7).

Acquired antimicrobial resistance (AMR) genes were identified using ResFinder version 4.1 (https://cge.food.dtu.dk/services/ResFinder-4.1/), applying a minimum nucleotide identity threshold of 90% and minimum gene coverage of 60% based on assembled contigs. All publicly available, high-quality *P. multocida* whole-genome sequences from the United States (*n* = 86) were retrieved from the Bacterial and Viral Bioinformatics Resource Center (BV-BRC) and analyzed for acquired AMR genes.

For phylogenetic analysis, publicly available complete genomes of *P. multocida* with associated metadata on host species and country of origin (*n* = 108) downloaded from the BV-BRC were analyzed together with the genomes from the United States after the removal of duplicate sequences (*n* = 194). Detailed information on all genomes included in the analyses is provided in Supplementary Table 1. Core-genome single nucleotide polymorphism (SNP) analysis were performed using kSNP4 (8), with the parameters “-k 17 -core -ML.” An SNP-based maximum likelihood tree was generated using FastTree (9), which is automatically implemented in the kSNP4 pipeline.

## 3 Results

The phenotypic antimicrobial susceptibility test results are summarized in Supplementary Table 3. All *P. multocida* isolates were susceptible to cefazolin, cefpodoxime, ceftiofur, chloramphenicol, enrofloxacin, florfenicol, and tulathromycin, whereas all were resistant to penicillin. Tetracycline resistance was detected in three bovine isolates (33.3%). Among these, one isolate (22-2477) exhibited a multidrug-resistant (MDR) phenotype, showing concurrent resistance to β-lactams (penicillin), aminocyclitols/aminoglycosides (spectinomycin), and tetracycline (tetracyclines). In addition, one feline isolate was resistant to amoxicillin-clavulanate (11.1%). Capsular and LPS genotyping showed that all isolates except one feline strain shared a common genotype across multiple host species, being classified as capsular type A and LPS type L3 (Table 1). In contrast, the feline isolate (22-2831) lacked a detectable capsule locus (Table 1); in this strain, the two genes typically flanking the capsule locus in *P. multocida, grxD* and *DUF441*, were found to be directly adjacent to each other. Additionally, this feline isolate was assigned to LPS type L1.

All five bovine isolates were assigned to ST1 under the Multi-Host MLST scheme and ST79 under the RIRDC MLST scheme (Table 1). In contrast, isolates recovered from non-bovine hosts were assigned to distinct sequence types, including ST374/ST622 from the deer, ST375/ST171 from the cat, ST373/ST623 from the pheasant, and ST312/ST519 from the rabbit (Table 1). Notably, ST622, ST623, ST374, ST373, and ST375 represented novel sequence types identified in this study and were subsequently submitted to the corresponding MLST databases. In addition, the allelic profiles defining ST622, ST623, and ST374 were newly identified and deposited in the respective MLST databases (Supplementary Table 4).

AMR gene analysis revealed that only bovine isolates from this study (*n* = 5) harbored AMR genes, and all exhibited an identical resistance gene profile consisting of *aph*(3′)-Ia, *aph*(6)-Id, *sul2*, and *tet*(H) (Table 1). No AMR gene was detected in isolates recovered from non-bovine hosts. To place these findings in a broader national context, publicly available *P. multocida* genomes from the United States (*n* = 86) were additionally screened for AMR genes (Supplementary Table 5). Among these, 42 isolates (48.8% of the isolates, 73% of bovine isolates) carried one or more AMR genes, and all AMR-positive isolates were of bovine origin.

**Figure 1:**
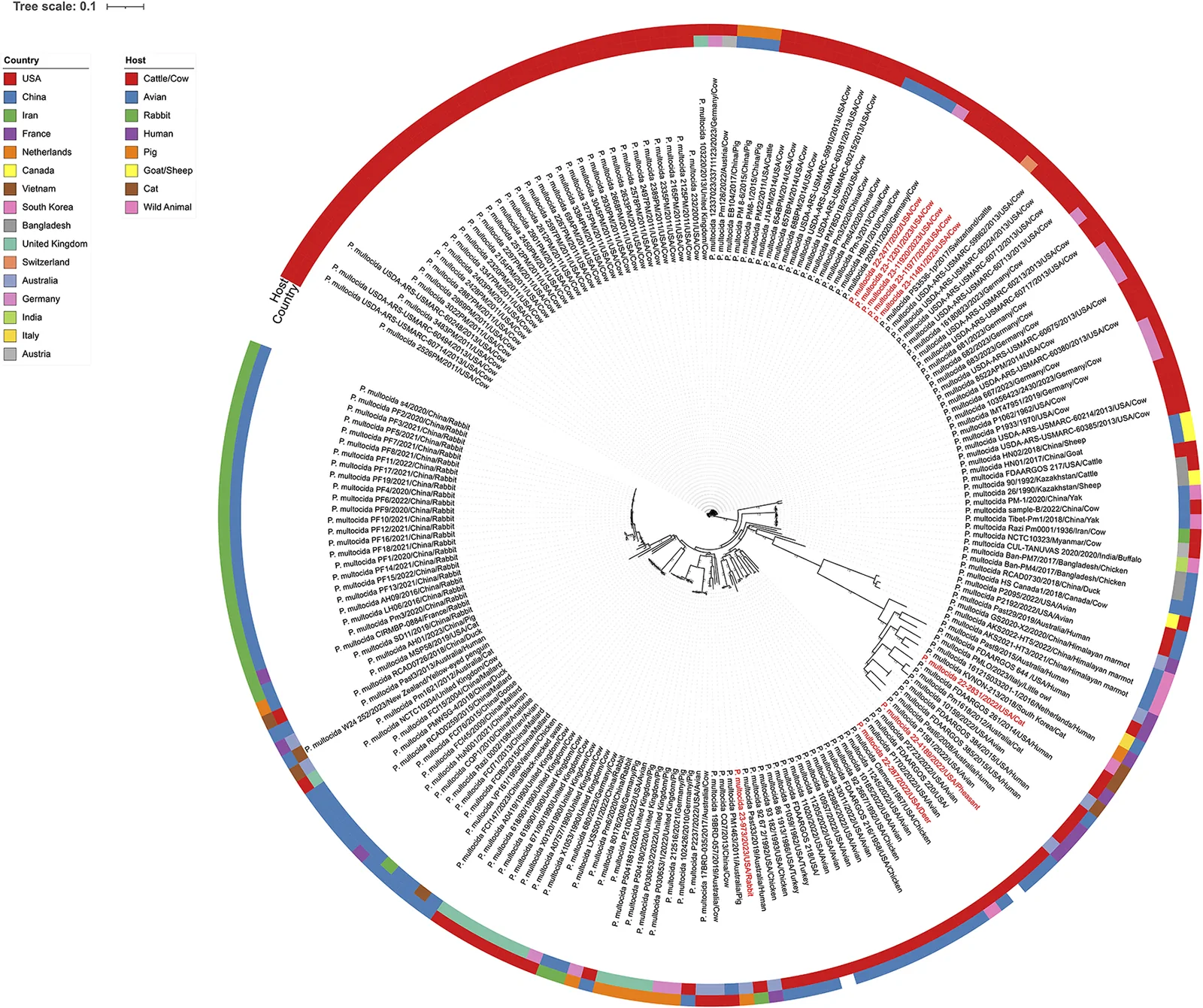
Core-genome SNP–based phylogenetic tree of *P. multocida* isolates. The tree was constructed using genomes sequenced in this study (*n* = 9), publicly available whole-genome sequences from the United States (*n* = 86), and publicly available complete genomes with associated metadata on host species and country of origin, excluding U.S. genomes (*n* = 108). The phylogeny was midpoint-rooted, and bootstrap support values based on 1,000 replicates are shown as percentages on selected branches. Isolates sequenced in this study are highlighted in red.

Virulence gene profiling of nine *P. multocida* isolates revealed that most virulence-associated genes were highly conserved across hosts (Supplementary Table 6). Core adhesins *fimA* and *ptfA* were detected in all isolates (9/9, 100%), whereas *tadD* (7/9, 78%), and *pfhA* (6/9, 67%) were present in the majority of isolates. In contrast, *hsf1* (2/9, 22%) and *hsf2* (5/9, 56%) were less frequently detected. Iron acquisition genes *exbB, exbD, fur*, and *hgbA* were universally present (9/9, 100%), together with *hgbB* (6/9, 67%) and *tbpA* (5/9, 56%). Outer membrane proteins *ompA, plpB*, o*mpH* (9/9, 100%), and *omp87* (8/9, 89%), sialidases *nanB* and *nanH*, and superoxide dismutases *sodA* and s*odC* (9/9, 100%) were also widely distributed. Overall, genes involved in adhesion, iron acquisition, serum resistance, and oxidative stress protection were highly conserved, regardless of the host origin. In core-genome SNP-based phylogenetic analysis, all five bovine isolates clustered together with the bovine isolates from United States and Germany (Figure 1). The feline isolate clustered with an isolate from South Korea within a clade that also included avian, feline, and human isolates. Notably, the deer and pheasant isolates grouped closely with avian isolates from the United States collected in 2022. In addition, the rabbit isolate, the only rabbit isolate from United States included in this analysis, clustered with a group composed predominantly of avian isolates from China and Vietnam, rather than with rabbit isolates from China.

## 4 Discussion

In this study, we characterized the phenotypic AMR, genomic features, and host-associated population structures of *P. multocida* isolates recovered from five animal species in Connecticut between 2022 and 2023. Despite the limited sample size, our findings provide insights into the host-linked genomic diversity and evolutionary dynamics of *P. multocida* circulating in domestic and wildlife hosts.

Capsular typing in the present study revealed a predominance of capsular type A (8/9), consistent with previous studies reporting that type A strains are commonly associated with infections across diverse host species (10, 11). Notably, one feline isolate lacked a detectable capsule locus, a finding that has also been described in previous studies reporting capsule-deficient *P. multocida* strains from both human and feline origins (11, 12). Although the biological implications of capsule loss remain unclear, such strains may exhibit distinct phenotypic characteristics, including enhanced biofilm formation and increased environmental persistence, suggesting a potential role in ecological adaptation rather than acute virulence (12). Given that *P. multocida* pathogenicity is influenced by multiple factors beyond the capsule, including lipopolysaccharide structure and accessory virulence determinants, the non-encapsulated isolate identified in this study may represent a host-adapted or alternative pathogenic lineage.

The antimicrobial susceptibility profiles observed in this study were generally consistent with previous reports describing low resistance rates among *P. multocida* isolates from wild and companion animals (13–16). However, all isolates in this study exhibited penicillin resistance, suggesting the emergence or localized circulation of penicillin-resistant lineages. Notably, the only multidrug-resistant isolate and all detected AMR genes were confined to bovine isolates, which displayed higher overall resistance levels, consistent with previous studies that reported elevated resistance among bovine *P. multocida* isolates (17). This pattern likely reflects greater antimicrobial exposure and selective pressure in livestock production systems. Although aminoglycoside, sulfonamide, and tetracycline resistance genes were detected in all bovine isolates, phenotypic resistance to spectinomycin was observed in only one isolate and tetracycline in three isolates. These findings suggest that the presence of resistance genes does not always translate into detectable phenotypic resistance and may be influenced by differences in gene expression or other regulatory factors. By contrast, isolates from non-bovine domestic and companion animals (cat, rabbit, and pheasant) and wild deer, showed no evidence of AMR determinants. A similar trend was reported in a recent study of *P. multocida* isolates from Australian companion animals and captive wildlife, where no antimicrobial resistance was detected in these hosts, although resistance to tetracyclines in bovine isolates and ampicillin in avian isolates was identified (16). Collectively, the findings highlight the host-associated differences in AMR within *P. multocida* and underscored the importance of targeted surveillance strategies across animal populations.

MLST analysis revealed a clear host-associated population structure. Consistent with large-scale genomic studies reporting both host-restricted and multi-host lineages (10, 18), ST79 was predominant among the bovine isolates in this study, in agreement with its recognition as a globally distributed, bovine-associated lineage (17) and its frequent detection among the bovine isolates in China (18). In contrast, isolates from non-bovine hosts were assigned to distinct sequence types, reflecting greater genetic heterogeneity. Supporting this observation, records from the PubMLST *P. multocida* database indicate that ST171 has been reported in turkey, chicken, and dog hosts in the United States and Australia, whereas ST519 and ST312 have been associated with turkey-derived isolates from the United States. These data suggest that the lineages identified in this study are not restricted to a single host species or geographic region. Moreover, the identification of multiple novel sequence types and allelic profiles further underscored the expanding genetic diversity of *P. multocida* and highlighted the need for genome-based surveillance across diverse hosts.

Analysis of virulence-associated genes showed that most virulence determinants were broadly conserved across host species, consistent with previous reports (10, 19). Although *tbpA* was initially reported in bovine isolates, its widespread detection in poultry, swine, rabbits, and other hosts in previous studies, along with the variable but non-host-restricted distribution of genes such as *hsf2* and *hgbB*, further support this conclusion (10, 19). In contrast, host-associated differences were reported when isolates were classified into 13 virulence gene profiles based on nine virulence-associated genes in the study by Ujvári et a*l*. (20). Similar patterns have also been reported in recent genomic studies, which have shown that although many virulence determinants are widely distributed among *P. multocida* isolates, variations in virulence gene combinations can contribute to host-associated population structure (16). In our dataset, *hsf1* was detected exclusively in bovine isolates, suggesting that while individual virulence genes are not strictly host-specific, variations in gene frequency may reflect ecological or epidemiological differences. Therefore, disease severity and clinical outcomes could possibly be influenced more by host factors or infection sites than by the presence of single virulence genes (19).

Core-genome SNP phylogenetic analysis revealed host-associated clustering, consistent with previous comparative genomic studies showing that *P. multocida* lineages tend to segregate by host species rather than by geography (10, 21). The clustering of isolates from the same host species was observed among bovine isolates in our dataset, regardless of the collection year or country, suggesting the persistence of relatively stable host-associated lineages. However, some isolates in our dataset showed phylogenetic associations across host species, including a rabbit isolate clustering with avian isolates from China and Vietnam and a deer isolate grouping with U.S. avian isolates. Similarly, recent genomic studies have reported phylogenetic groupings that include isolates from diverse host species, indicating potential ecological overlap or cross-host transmission; notably, comparative genomic analyses have also shown that while human infections are caused by a relatively narrow subset of *P. multocida* strains, companion animals such as cats and dogs harbor a much broader diversity of strains and may serve as reservoirs for zoonotic transmission (11, 16, 18). Together, these observations suggest that while host-associated lineages are present, occasional host switching or circulation among multiple hosts may contribute to the global population structure of *P. multocida*.

In conclusion, our study provides a phenotypic and genomic characterization of *P. multocida* isolates recovered from multiple domestic and wildlife hosts in Connecticut. Host-associated patterns were observed, with antimicrobial resistance largely confined to bovine-associated lineages, while isolates from non-bovine domestic, wildlife, and companion animals remained largely susceptible and genetically diverse. The detection of previously unreported sequence types highlights the contribution of under-sampled hosts to the overall population structure of *P. multocida*. Genome-wide phylogenetic analyses further support a role for host adaptation in shaping lineage diversification. However, given the limited number of non-bovine isolates included in this study, these observations should be interpreted cautiously and warrant confirmation using larger datasets from diverse hosts. Together, these findings underscore the value of integrated genomic surveillance across diverse animal hosts and provide a framework for improved epidemiological monitoring and One Health–informed disease control strategies.

## Data Availability

All sequence data are publicly available in GenBank under BioProject PRJNA1381296.
